# Rapid Photoinduced Single Cell Detachment from Gold Nanoparticle-Embedded Collagen Gels with Low Denaturation Temperature

**DOI:** 10.3390/polym12010213

**Published:** 2020-01-15

**Authors:** Chie Kojima, Misaki Nishio, Yusuke Nakajima, Takeshi Kawano, Kenji Takatsuka, Akikazu Matsumoto

**Affiliations:** 1 Department of Applied Chemistry, Graduate School of Engineering, Osaka Prefecture University, 1-1 Gakuen-cho, Naka-ku, Sakai, Osaka 599-8531, Japan; szb02092@edu.osakafu-u.ac.jp (M.N.); swb02101@edu.osakafu-u.ac.jp (Y.N.); matsumoto@chem.osakafu-u.ac.jp (A.M.); 2 Research & Development Division, Optical Research Laboratory, Nikon Corporation, 471, Nagaodai-cho, Sakae-ku, Yokohama, Kanagawa 244-8533, Japan; Takeshi.Kawano1@nikon.com (T.K.); Kenji.Takatsuka1@nikon.com (K.T.)

**Keywords:** collagen, gold nanoparticle, telopeptide, denaturation, photoinduced, cell detachment

## Abstract

Cell Separation is important in various biomedical fields. We have prepared gold nanoparticle (AuNP)-embedded collagen gels as a visible-light-responsive cell scaffold in which photoinduced single cell detachment occurs through local thermal denaturation of the collagen gel via the photothermal effect of AuNP. Physicochemical properties of collagen materials depend on the origin of the collagen and the presence of telopeptides. In this study, we prepared various AuNP-embedded collagen gels by using different collagen materials with and without the telopeptides to compare their thermal denaturation properties and photoinduced single cell detachment behaviors. Cellmatrix type I-C without telopeptides exhibited a lower denaturation temperature than Cellmatrix type I-A and Atelocell IAC, as examined by Fourier transform infrared (FTIR) spectroscopy, rheological analysis, and sol–gel transition observation. Three-dimensional (3D) laser microscopic imaging revealed that collagen fibers shrank in Cellmatrix type I-A upon heating, but collagen fibers disappeared in Cellmatrix type I-C upon heating. Cells cultured on the Cellmatrix type I-C-based AuNP-embedded collagen gel detached with shorter photoirradiation than on the Cellmatrix type I-A-based AuNP-embedded collagen gel, suggesting that collagen gels without telopeptides are suitable for a photoinduced single cell detachment system.

## 1. Introduction

**Figure 1:**
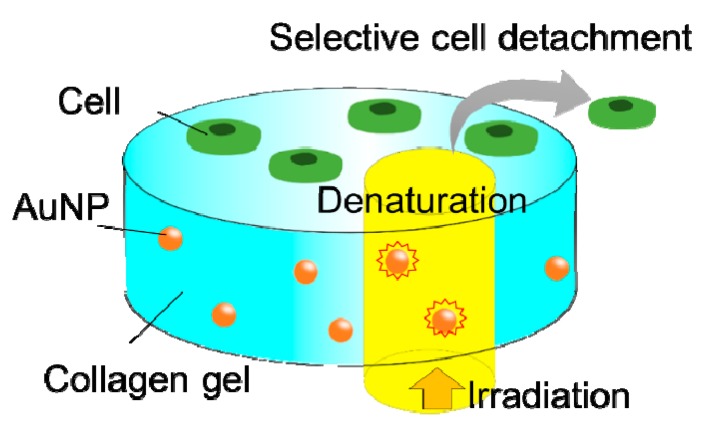
Schematic illustration of photoinduced cell detachment.

**Figure 2:**
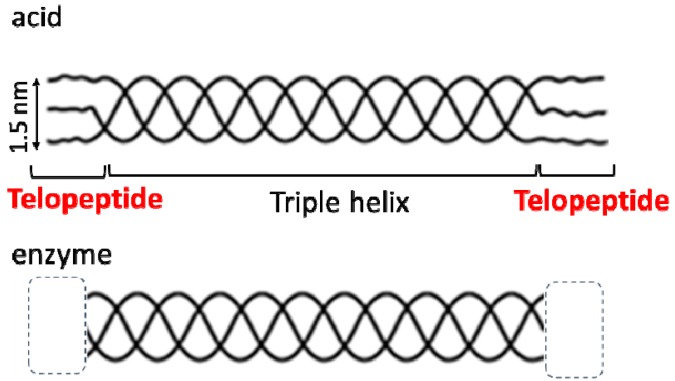
Collagen materials obtained by acid and enzyme treatment.

Cell separation is crucial to various biomedical applications, such as cell engineering and regenerative medicine [1,2,3]. Because fluorescence-activated cell sorting (FACS) cannot be applied in spatiotemporal cell separations, image-guided cell sorting systems have been developed [4,5]. We have constructed a visible-light-induced single cell detachment system from gold nanoparticle (AuNP)-embedded collagen gels by using a microscope equipped with a laser irradiation system [6,7]. Collagen has the thermal denaturation properties and AuNPs have the photothermogenic property due to localized surface plasmon resonance [8,9]. Thus, the AuNP-embedded collagen gel can work as a visible-light-responsive cell scaffold. Previously, single cells were selectively detached from AuNP-embedded collagen gels by the visible laser irradiation (Figure 1). Because visible light presents low optical damage to living cells, this is a cell-friendly separation system [6,7]. A key aspect in this system is the local thermal denaturation of the collagen gel induced by the photothermal effect of AuNP. Collagen is composed of glycine-proline-(hydroxy)proline [Gly–Pro–Pro(Hyp)] repeats, which form a triple helix that assembles at low temperature and degrades upon heating [10,11,12]. The thermal denaturation properties of collagen depend on the content of hydroxyproline and the presence of telopeptides located at both termini of the triple helix region (Figure 2). The hydroxyproline content of collagen differs between species. For example, porcine collagen containing more Hyp has a higher denaturation temperature than that of fish collagen [12]. Collagen preparation procedures affect the presence of telopeptide. Acid-extracted collagens maintain the telopeptide regions, but enzyme-treated collagens do not. It is reported that the telopeptide affects the crosslinking as well as the immunogenicity of the collagen [13,14]. Therefore, the physicochemical properties of collagen materials largely depend on the collagen origin and the collagen preparation procedures [11,12,13].

In this study, AuNP-embedded collagen hydrogels were prepared using various collagen materials derived from different origins, with and without telopeptides. The thermal denaturation properties of these collagen hydrogels with and without AuNPs were examined by Fourier transform infrared (FTIR) spectroscopy, rheological analysis, sol–gel transition observations and three-dimensional (3D) laser microscopic imaging. Finally, model cells cultured on these AuNP-embedded collagen gels were detached with short photoirradiation to elucidate the relationship between the denaturation properties of the collagen gel and the cell detachment process.

## 2. Materials and Methods

### 2.1. Preparation of AuNP-Embedded Collagen Gels

Cellmatrix type I-A and I-C (3 mg/mL) and Atelocell IAC-50 (5 mg/mL) were purchased from Nitta Gelatin Inc. (Osaka, Japan) and KOKEN Co., Ltd. (Tokyo, Japan), respectively. The properties of these collagen materials are listed in Table 1. AuNPs with 50 nm in average diameter were prepared by the seeded growth method using hydroquinone, as previously described [15]. AuNP-embedded collagen gels were prepared according to the manufacturer’s instructions, as previously described [6]. Briefly, the AuNP solution (750 µM) was prepared by centrifugation (25 °C, 4480 g) for 20 min after a wash with ultrapure water. For the cell detachment experiment, the collagen solution (0.56 mL), the AuNP solution (0.7 mL), HCl (1 mM, 0.42 mL), the 10× concentrated medium (0.21 mL) and the regenerative buffer solution (0.21 mL) were mixed in this order on ice. The mixed solution (50 µL) was poured into a 96-well plate and incubated at 37 °C for 30 min. The collagen gels without AuNPs were prepared by replacing the AuNP solution with ultrapure water.

### 2.2. Characterization

The sol–gel transition temperature was measured as follows: The collagen solution (294 µL of Cellmatrix type I-A or IC or 177 µL of Atelocell IAC mixed with 117 µL of 1 mM HCl), the AuNP solution (210 µL), the 10× concentrated medium (63 µL) and the regenerative solution (63 µL) were mixed, in this order, in a tube on ice. The mixture was incubated at 37 °C for 30 min to obtain the AuNP-embedded collagen gels. A 4 mm diameter plastic ball (~2 g) was placed on the gel. The gel was heated from 38 °C in 2 °C intervals. Gel images were obtained after 15 min incubation at each temperature. The sol–gel transition temperature was determined as the temperature at which the ball sank to the bottom. As for the FTIR spectroscopic analysis, the hydrogels were incubated at the predetermined temperature (37~60 °C) for 15 min, and they were then frozen using liquid nitrogen. After freeze-drying, the residues were crushed to prepare KBr pellets. These FTIR spectra were recorded on FT-IR4600 spectrometer (Jasco Inc., Tokyo, Japan), normalized to the signal around 1455 cm^−1^. Three independent experiments were performed for the sol–gel transition temperature measurements and the FTIR spectroscopic analysis.

Rheological analysis was performed using a dynamic rheometer (HAAKE MARSIII, Thermofisher Scientific Inc., Waltham, MA, USA). The collagen solution (0.98 mL of Cellmatrix type I-A or I-C, or 588 µL of Atelocell IAC mixed with 392 µL of 1mM HCl), the AuNP solution (0.7 mL), the 10× concentrated medium (0.21 mL), and the regenerative solution (0.21 mL) were mixed in this order on ice. The mixed solutions were quickly placed between parallel plates with a 25 mm diameter and a gap of 1.0 mm. The plates were retained at 37 °C for 30 min, and then heated at 1 °C/min to 60 °C. The dynamic moduli were measured by applying an oscillatory shear strain under constant strain amplitude of 0.01 at 6.28 rad/s.

Collagen fibers of AuNP-embedded collagen gels in phosphate buffered saline (PBS) were observed at different temperatures with a 3D measuring laser microscope OLS5000 or OLS4100 (Olympus Co., Tokyo, Japan) equipped with a sample heating unit (Shimadzu Co., Kyoto, Japan). The temperature was monitored using a temperature data logger equipped with a thermocouple (Shimadzu Co., Kyoto, Japan).

### 2.3. Laser-Induced Cell Detachment

Laser-induced cell detachment was carried out as previously described, except with adjustments in irradiation time [6,7]. HeLa cells (6000 cells/well) were seeded onto AuNP-embedded collagen gels in 96-well plates. After 24 h incubation, the cells were washed thrice with PBS. After adding PBS (10 µL) to the gel, cells were observed under an inverted fluorescence microscope (ECLIPSE Ti-U, Nikon Co., Tokyo, Japan) using imaging software (WraySpect, Wraymer Inc., Osaka, Japan). The microscope was also equipped with a laser irradiation system (Sigmakoki Co., Ltd, Tokyo, Japan), micromanipulator (NT-88-V3MSH, Narishige Group, Tokyo, Japan), and microinjector (IM-11-2, Narishige Group). The laser (532 nm, 50 mW) was used to irradiate the target cells for 2 s with 10× Plan-Fluor (Nikon Co.) as the objective lens. The laser-irradiated cells were aspirated using the micromanipulator system with a 30-µm glass capillary. Cells were observed under the microscope before and after the photoirradiation and aspiration. The cell detachment experiment was performed more than 10 times to obtain the cell detachment efficiency.

## 3. Results

### 3.1. Thermal Denaturation of Different Collagen Gels

**Figure 3:**
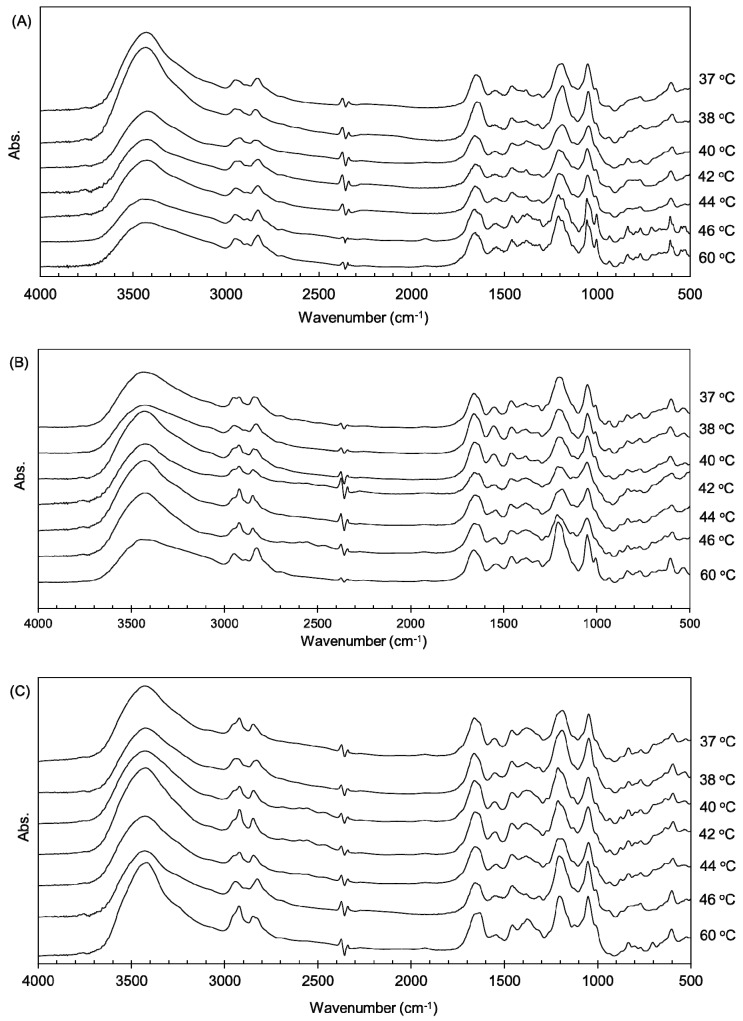
Fourier transform infrared (FTIR) spectra at different temperatures of AuNP-embedded collagen gels made from Cellmatrix type I-A (**A**), Atelocell IAC (**B**), and Cellmatrix type I-C (**C**).

**Figure 4:**
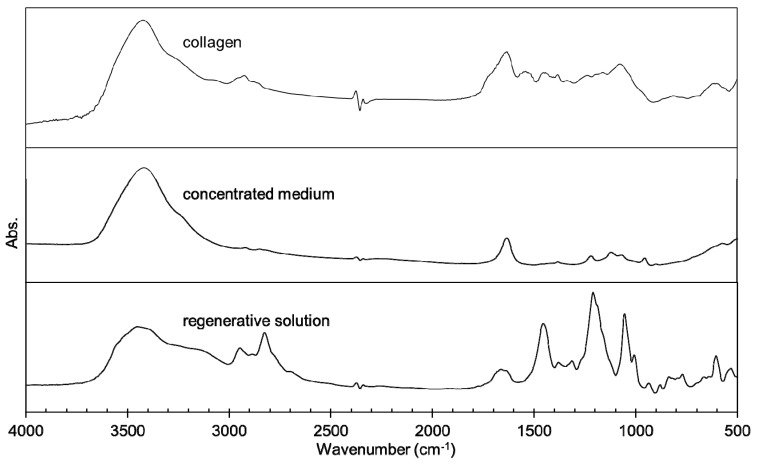
FTIR spectra of the collagen solution, the concentration medium, and the regenerative solution.

**Figure 5:**
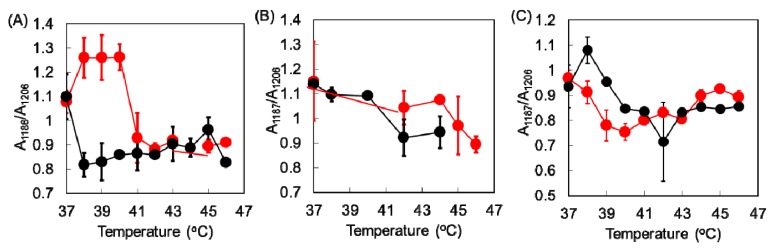
Ratio of amide III signals (1186 cm^−1^/1206 cm^−1^) of collagen gels with and without AuNPs (red and black) as a function of temperature. (**A**) Cellmatrix type I-A, (**B**) Atelocell IAC, and (**C**) Cellmatrix type I-C.

Three types of collagen materials, Cellmatrix type I-A, Atelocell IAC, and Cellmatrix type I-C, were used for collagen gel preparation with and without AuNPs. Cellmatrix type I-A and Atelocell IAC contain telopeptide regions, but Cellmatrix type I-C does not. The thermal denaturation properties of these different collagen materials were examined and compared. First, the higher order structure of the AuNP-embedded collagen gels was examined using FTIR spectroscopy. The characteristic collagen signals derived from amide A (3328 cm^−1^), amide B (3084 cm^−1^), amide I (1655 cm^−1^), amide II (1555 cm^−1^), and amide III (1240 cm^−1^) are known [16,17]. These signals were observed in the AuNP-embedded collagen gels (Figure 3). The amide III signal is a complex vibrational mode derived from the combination of C–N stretching and N–H in-plane bending. Thus, different discrete absorptions in amide III were observed around 1200 cm^−1^ [16]. The denaturation by heating of the collagen triple helix structures formed in collagen can be monitored by taking the ratio of amide III/1455 cm^−1^ [17,18]. The average values of 1235 cm^−1^/1455 cm^−1^ of Cellmatrix type I-A, Atelocell IAC, and Cellmatrix type I-C before heating were 1.4, 1.3 and 1.2, respectively, and no obvious changes of the ratio were observed after heating. Figure 4 shows that the signals of additive components in the concentrated medium and the regenerative solution overlapped the collagen characteristic signals in the FTIR spectra. It is possible that the change in collagen signals was not detected over the strong overlapping signals of other components. However, the peak patterns derived from amide III (around 1200 cm^−1^) changed upon heating (Figure 3). The signal ratio of 1186 to 1206 cm^−1^ was plotted against temperature (Figure 5). The signal ratio of AuNP-embedded Cellmatrix type I-A, Atelocell IAC, and Cellmatrix type I-C dropped at 41, 45, and 39 °C, respectively, whereas these intensities without AuNP dropped at 38, 42, and 40 °C, respectively. These results suggest that the higher order structure of the collagen gels was possibly changed by heating. Our results also indicate that the embedded AuNP affected the higher order structure of Cellmatrix type I-A and Atelocell IAC, but not Cellmatrix type I-C.

**Figure 6:**
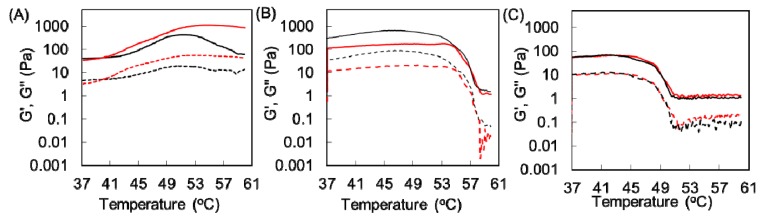
Rheological analysis of different collagen gels with and without AuNP (red and black). (**A**) Cellmatrix type I-A, (**B**) Atelocell IAC, and (**C**) Cellmatrix type I-C. G’ and G’’ are shown as solid and dotted lines, respectively.

We next performed rheological analysis of these collagen gels. Figure 6 shows that the elastic modulus (G’) of AuNP-embedded Cellmatrix type I-C and Atelocell IAC gels decreased above 45 and 54 °C, respectively. The viscous modulus (G”) also dropped at the same temperature. These results suggest that the hydrogel state of these collagen gels changed at these temperatures. On the other hand, the G’ value of AuNP-embedded Cellmatrix type I-A increased upon heating until around 55 °C and then slightly decreased above this temperature. The thermal response of Cellmatrix type I-A was much different from that of the other collagen materials. Because the results of rheological analysis were similar for the collagen gels without AuNPs and those with AuNPs, the difference was observed because of the collagen materials.

**Figure 7:**
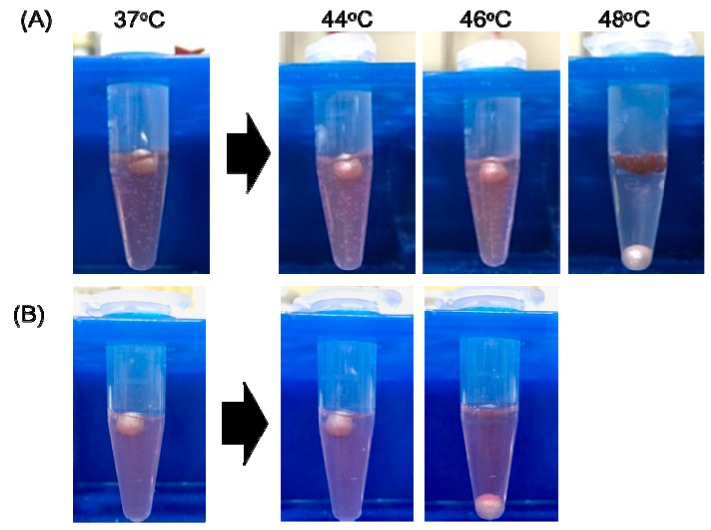
Sol–gel experiment of AuNP-embedded collagen gels. (**A**) Cellmatrix type I-A and (**B**) Cellmatrix type I-C.

We also examined the sol–gel transition temperature of the collagen gels with and without AuNPs. The gel state is determined when the ball is on the gel, and the sol state is determined when it sinks to the bottom of the tube. The AuNP-embedded collagen gels made from Cellmatrix type I-A and Cellmatrix type I-C form a hydrogel below 46 and 44 °C, respectively, and these gels changed at 48 and 46 °C, respectively (Figure 7). Red-colored AuNPs were distributed in the Cellmatrix type I-C solution upon heating, indicating that this collagen gel had dissolved. On the other hand, AuNPs were condensed in the Cellmatrix type I-A-based gel after heating, suggesting that this collagen gel did not dissolve. The experiments were performed on the other collagen gels with and without AuNPs to estimate the sol–gel transition temperatures listed in Table 2. The sol–gel transition temperatures for each collagen gel were the same with and without AuNPs. Cellmatrix type I-C had a lower transition temperature than Cellmatrix type I-A or Atelocell IAC.

### 3.2. Thermal Changes of Collagen Fibers in Collagen Gels Containing AuNPs

**Figure 8:**
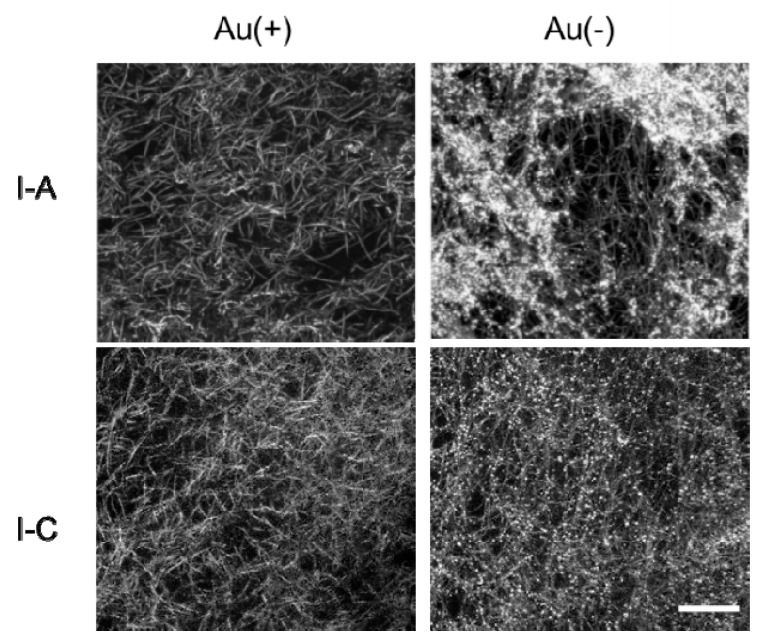
3D laser microscopic images of collagen gels made from Cellmatrix type I-A and Cellmatrix type I-C, with and without AuNPs. Bar: 20 μm.

**Figure 9:**
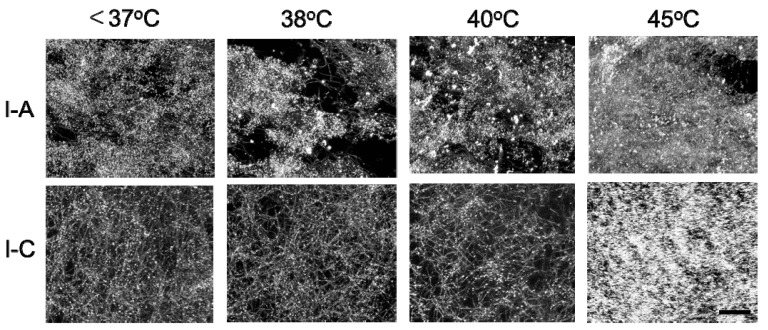
3D laser microscopic images at different temperatures of AuNP-embedded collagen gels made from Cellmatrix type I-A and Cellmatrix type I-C. Bar: 20 μm.

The collagen fibers in Cellmatrix type I-A and Cellmatrix type I-C gels were observed with 3D laser microscopic imaging. Fibrous structures corresponding to collagen fibers were observed in all the images, and bright dots corresponding to AuNPs were observed in AuNP-embedded collagen gels (Figure 8). The AuNP-embedded collagen gels were observed during heating to investigate thermal changes in structure. The thermal changes of the collagen gels varied (Figure 9). The Cellmatrix type I-A gel began shrinking above 38 °C. On the other hand, the Cellmatrix type I-C gel seemed to be unchanged around 40 °C, but the collagen fibers suddenly disappeared at around 45 °C to induce drastic movement in the AuNP dots. This observation was consistent with those found in our rheological analyses (Figure 6) and sol–gel transition experiment (Figure 7).

### 3.3. Selective Cell Detachment from Various AuNP-Embedded Collagen Gels

**Figure 10:**
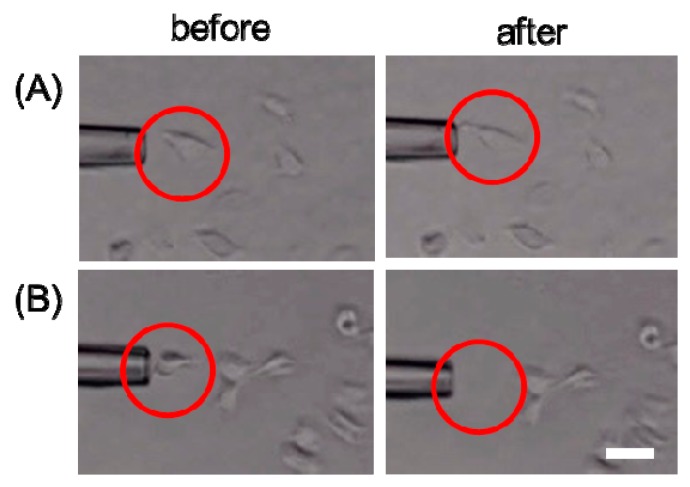
Single cell detachment from AuNP-embedded collagen gels made from Cellmatrix type I-A (**A**, AuCol-IA) and Cellmatrix type I-C (**B**, AuCol-IC) by photoirradiation for 2 s and subsequent aspiration. The photoirradiated cells are marked with a circle. Bar. 30 μm.

We have previously used Cellmatrix type I-A for preparing AuNP-embedded collagen gels from which photoirradiated cells were selectively detached with 10-s photoirradiation and subsequent aspiration [6,7]. In this study, AuNP-embedded collagen gels were prepared from Cellmatrix type I-A and Cellmatrix type I-C with high and low denaturation temperatures, respectively, for single cell detachment by photoirradiation for 2 s. The photoirradiated cells were selectively detached from the Cellmatrix type I-C-based AuNP-embedded collagen gel (AuCol-IC), but not from the Cellmatrix type I-A-based AuNP-embedded collagen gel (AuCol-IA), and the unirradiated cells were not detached from AuNP-embedded collagen gels (Figure 10). We repeated the same cell detachment experiment over 10 times. Photoinduced cell detachment succeeded in 46% of attempts on AuCol-IC but only in 13% on AuCol-IA. Of the photoirradiated cells, 85% were moved on AuCol-IC after aspiration but only 38% on AuCol-IA. These results indicate that cells can detach with shorter photoirradiation from AuCol-IC than from AuCol-IA. The short-term photoirradiation time is advantageous for reducing cell damage as well as high throughput selection. For this photoirradiation system, optimization of parameters, such as irradiation area and laser power, are necessary for practical application.

## 4. Discussion

The thermal denaturation behaviors of the three types of collagen gels with and without AuNPs, which were examined by different analyses, are summarized in Table 2. The trend in the denaturation temperature results was the same as that of the sol–gel results: Cellmatrix type I-C showed a lower denaturation temperature than Atelocell IAC or Cellmatrix type I-A, irrespective of the presence of AuNP in the collagen gels. The denaturation temperature estimated from the sol–gel experiments and rheological analysis were higher than the denaturation temperature estimated from FTIR spectrometry. This suggests that the change in the higher order collagen structure was the initial change in the denaturation process. Both sol–gel and hydrogel transition temperatures then affected the collagen fibril networks as macroscopic changes. The thermal properties of collagen materials were already reported by other groups [11,12,13,17,18,19]. Although the denaturation temperature was dependent in the sample conditions such as the water content, our results are not inconsistent with previous reports.

Our results indicate that cells were detached with shorter photoirradiation from AuCol-IC with lower denaturation temperature than from AuCol-IA. It is known that reactive oxygen species (ROS) is generated from AuNP after the irradiation of UV, X-ray and pulse laser with high energy [20,21]. Because a continuous wave (CW) green laser was used in our experiment, it is unlikely that ROS was generated from AuNP in our system. Our previous report indicated that ROS was not detected in photoirradiated cells on the AuNP-embedded collagen gels [7]. Besides, denaturation temperature of AuNP-embedded collagen gels was well correlated with the cell detachment behaviors. Thus, the local thermal denaturation of the collagen gel induced by the photothermogenic property of AuNPs is crucial for the photoinduced selective cell detachment. Our previous reports also indicate that any significant cytotoxicity was not observed in the cells cultured on the AuNP-embedded collagen gels before and after the photoirradiation [6,7], suggesting that this is a cell-friendly cell separation system.

## 5. Conclusions

In this study, we examined the thermal denaturation behaviors of various AuNP-embedded collagen gels with FTIR spectroscopy, rheological analysis, sol–gel observations, and 3D microscopic imaging. The Cellmatrix type I-C-based collagen gel without telopeptides denatured at a lower temperature than those with telopeptides (Cellmatrix type I-A and Atelocell IAC), regardless of the presence of AuNPs. Our results indicate that the secondary structure in AuNP-embedded collagen gels made from Cellmatrix type I-C changed above 39 °C and the collagen fibers as well as the collagen gel dissolved at around 45 °C. In contrast, the collagen fibers in the AuNP-embedded collagen gels made from Cellmatrix type I-A shrank upon heating. Photoirradiated cells could be selectively detached from AuNP-embedded collagen gels made from Cellmatrix type I-C after only 2 s of photoirradiation. Collagen gels without telopeptides are suitable for single cell separation system by visible light irradiation, presenting a cell-friendly image-guided single cell separation method.

## Figures and Tables

**Table 1:** Collagen used in this study.

Collagen	Origin	Preparation
**Cellmatrix Type I-A**	porcine tendons	acid
**Atelocell IAC-50**	bovine dermis	acid
**Cellmatrix Type I-C**	porcine skin	pepsin

**Table 2:** Denaturation temperatures estimated from FTIR spectra, rheological analysis, and the sol–gel experiment.

Collagen	Telopeptide	Au	FT-IR (°C)	Rheology (°C)	Sol–gel (°C)
Cellmatrix Type I-A	+	-	38	51 ^1^	50 ± 2
		+	41	55 ^1^	50 ± 2
Attelocell IAC	+	-	42	48 ^2^	52 ± 0
		+	45	54 ^2^	52 ± 0
Cellmatrix Type I-C	+	-	40	43 ^2^	46 ± 0
		+	39	45 ^2^	46 ± 0

^1^ The temperature of G’_max_, ^2^ The temperature at a 5% decrease from G’_max_.
